# A numerical simulation method for pressure drop and normal air velocity of pleated filters during dust loading

**DOI:** 10.1371/journal.pone.0282026

**Published:** 2023-02-21

**Authors:** Guangping Teng, Guoqing Shi, Jintuo Zhu, Jiamin Qi

**Affiliations:** 1 School of Safety Engineering, China University of Mining and Technology, Xuzhou, China; 2 School of Safety and Management Engineering, Hunan Institute of Technology, Hengyang, China; 3 State Key Laboratory of Coal Resources and Safe Mining, China University of Mining and Technology, Xuzhou, China; NUST: National University of Sciences and Technology, PAKISTAN

## Abstract

Pressure drop is an important indicator that affects the filtration performance of the pleated filter, and the deposition of dust particles within the pleats is crucial to the evolution of the pressure drop. In this study, the pressure drop during PM_10_ loading process was investigated for a series of V-shaped and U-shaped filters with a pleat height of 20 mm and different pleat ratios (the ratio of pleat height to pleat width: α = 0.71–3.57). In the numerical simulations, numerical models suitable for different pleated geometries were obtained through experimental verification on the local air velocity. Then, assuming that the dust cake thickness is proportional to the normal air velocity of the filters, the variation of the pressure drop with the dust deposition is derived by means of successive numerical simulations. This simulation method saved a significant amount of CPU time required for the growth of dust cake. It was found that the relative average deviations between experimental and simulated pressure drops were 3.12% and 1.19% for V-shaped and U-shaped filters, respectively. Furthermore, it was found that under the same pleat ratio and the mass of dust deposition per unit area, both the pressure drop and unevenness of normal air velocity of the U-shaped filter were lower than the V-shaped filter. Therefore, the U-shaped filter is recommended due to its better filtration performance.

## 1 Introduction

V-shaped and U-shaped filters, which have the advantages of simple structure and easy processing, are extensively adopted in HVAC systems, air purifiers and other fields for the purpose of reducing the concentration of particulate matter in our living environment and promoting air quality. Compared with the flat filter, the pleated filter has a larger filtration area and dust capacity [1–3], a lower filtration velocity and a smaller pressure drop under the same size [4–6]. However, it needs to be replaced regularly because the pressure drop will increase and the air volume will decrease with the continuous deposition of dust particles. That is to say, the service life of the filter is limited.

The pleating of the filter media can lead to the change of flow field [7–10], resulting in the uneven deposition of dust particles which in turn affects the evolution of the pressure drop [11–13]. Therefore, the flow field of the pleated filters and the uneven deposition of dust particles are to be investigated. Many studies have used numerical simulations to predict the pressure drop of the unloaded pleated filters [4, 7, 12, 14–20]. However, it was found that the velocity fields varied considerably when the simulated pressure drop was essentially the same for different models [7, 14, 16, 17], so further validation of the velocity field is required. Some studies have validated the velocity field by means of hot-wire anemometry (HWA) [14, 16] and micro-particle image velocimetry (μ-PIV) [7, 17]. HWA was used as a tool to generate local quantitative air velocity data to verify the velocity fields generated by the numerical simulations. In this paper, a hot-wire anemometer was used to select suitable models.

Variations in the flow field due to the pleated structure of the cartridge can lead to uneven deposition of dust particles [10–13]. Bourrous et al. [21] observation of the formation of dust cake by camera, but only at the pleat inlet and not inside the pleats. The small space inside the pleats makes it difficult to observe the dust deposition directly and requires the use of numerical simulation method. Fotovati et al. [12, 22] and Hettkamp et al. [19] modelled the growth of dust cake on pleated filters by defining user defined functions. However, this requires a significant amount of CPU time to track the trajectory of the particles and simulate the growth of dust cake. Saleh et al. [18] and Bourrous et al. [21] found that the inertia of the particles can be ignored at small Stokes numbers. In this study, the deposition of PM_10_ was investigated and the dust cake thickness can be considered to be proportional to the normal air velocity of filter (NAVF) due to the small Stokes number of the particles in the experiment. The pressure drop of the pleated filters during dust loading can be derived by means of successive numerical simulations. This simulation method saved a significant amount of CPU time required for the growth of dust cake.

In this study, a numerical simulation method for dust particle deposition on the V-shaped and U-shaped filters at small Stokes numbers was presented and validated experimentally, which can be used to predict the service life and optimize the design of the pleated structure of the filters.

## 2 Materials and methods

### 2.1 Experimental materials

**Fig 1:**
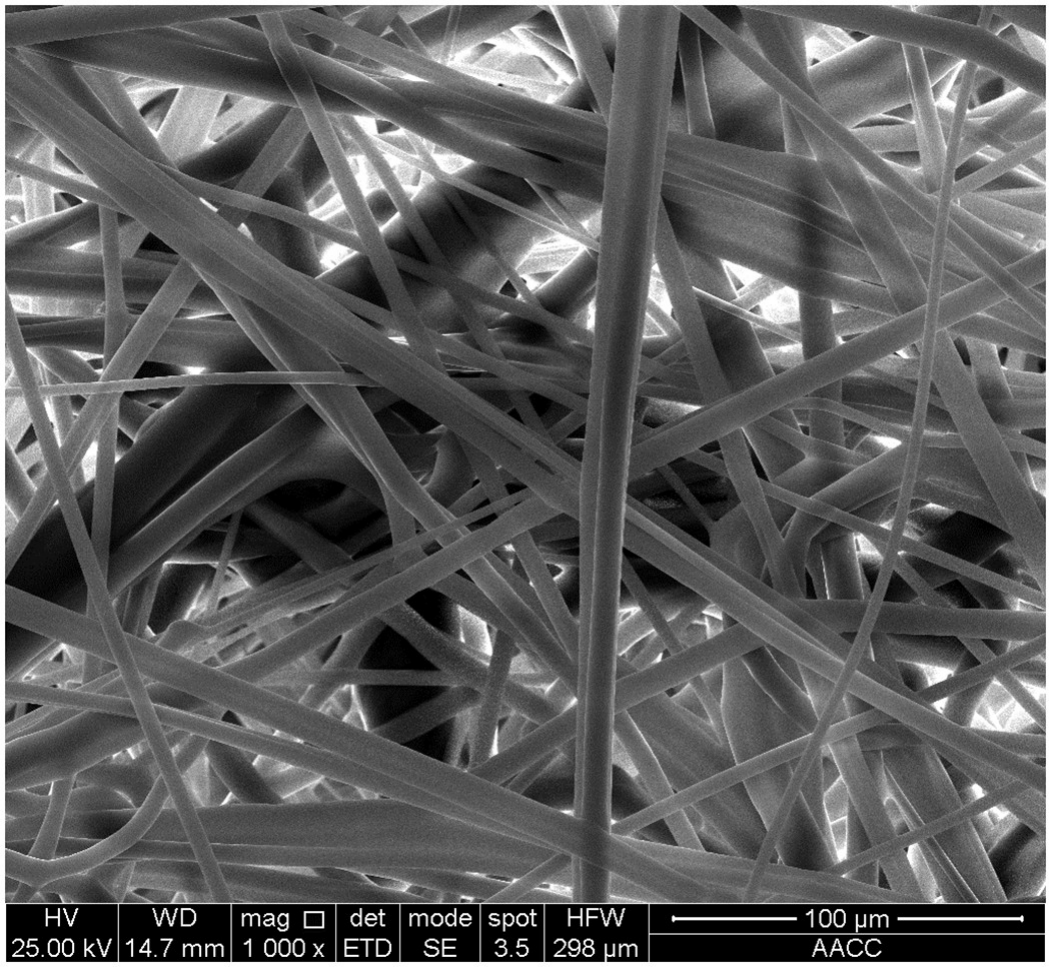
SEM image of filter media at the magnification of 1,000.

In this experiment, the filter media of polypropylene Microfiber (Henan Aklly Filter Engineering Co., Ltd., Xinxiang, China) was used. It had a filter grade of E10 (classified according to EN 1822-1-2019 [23]), a thickness of 0.5 mm, a grammage of 110 g/m^2^, a permeability of 9.5810×10^−12^ m^2^ and a porosity of 0.84. The scanning electron microscope (SEM, VEGA, TESCAN, Czech) image of the surface of the filter media is displayed in Fig 1 where the fiber presents a three-dimensional disordered spatial network structure. According to statistical calculation of the fiber diameter in the image, the average diameter of the filter fiber was around 4.6±0.3 μm.

**Fig 2:**
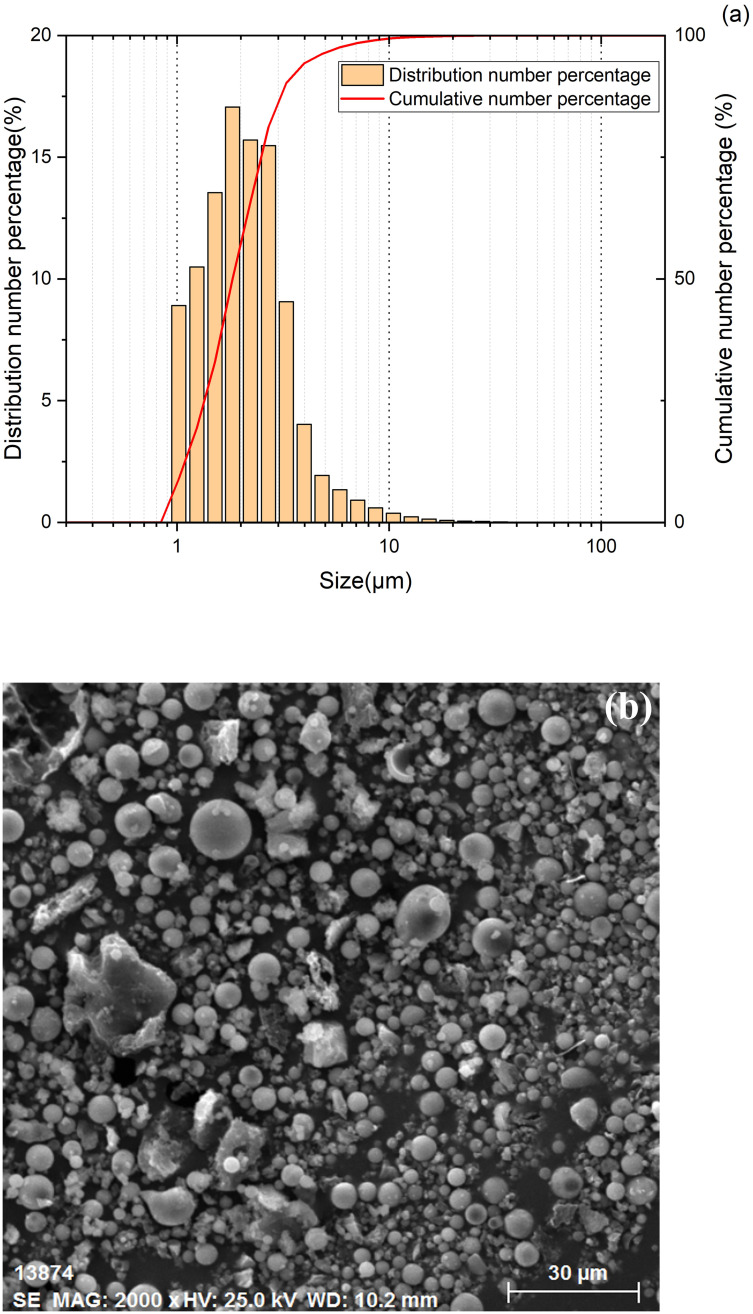
Particle size distribution and ESEM image of the fly ash (a) Particle size distribution, (b) ESEM image.

The dust used in this experiment was 400-mesh fly ash with a density of 620 kg/m^3^, which was dried in a drying box at a constant temperature of 100°C for 5 h. The distribution of different-sized dust particles was measured with the aid of a laser particle size analyzer (Winner2000, Jinan Winner Particle Instrument Stock Co., Ltd., Jinan, China), and the results are displayed in Fig 2(a). The results reveal that the count average particle size and count median particle size of the dust particles are 2.39 μm and 1.98 μm, respectively. Fig 2(b) gives the image of the fly ash observed with the environmental scanning electron microscope (ESEM, Quanta 250, FEI, the USA). It can be seen from the image that most of the dust particles are approximately spherical.

**Fig 3:**
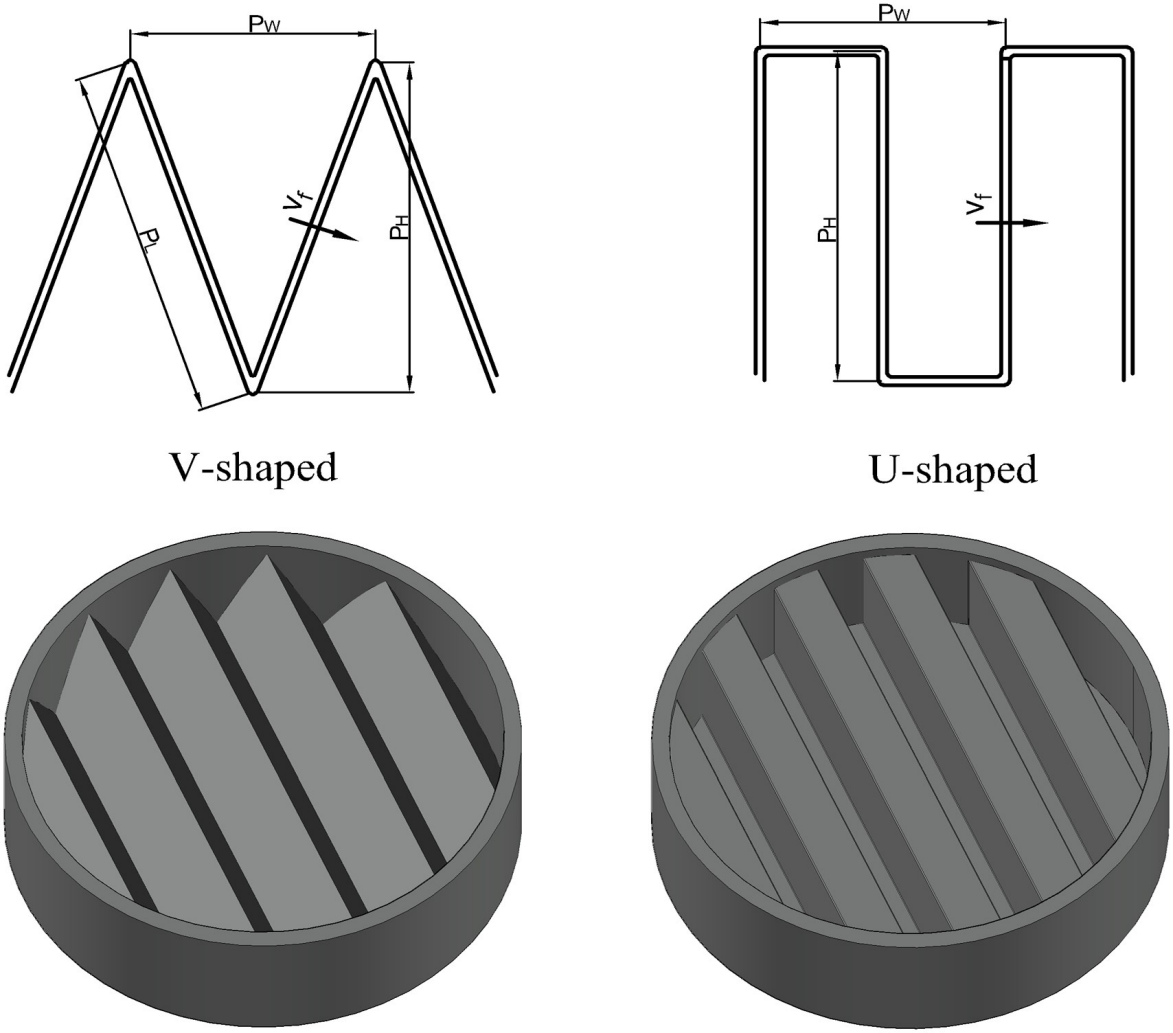
Geometric structures of the pleats.

In the experiment, the pleated filters were self-made. Their outer frame was a transparent polymethyl methacrylate ring with an outer diameter of 150 mm, an inner diameter of 140 mm and a height of 25 mm. The geometric structures of the V-shaped and U-shaped filters are shown in Fig 3. The number of pleats of the self-made filter were 5, 10, 15, 20 and 25, respectively, and the pleat height was 20 mm. The filter media was fixed and sealed with the outer frame using hot-melt adhesive. Specific parameters of the self-made filter are given in Table 1 where the pleat ratio of the flat filter is deemed to be 0.

**Table 1:** Parameters of the self-made pleated filter.

Pleat ratio (α)	0	0.71	1.43	2.14	2.86	3.57
Number of pleats (N)	/	5	10	15	20	25
Pleat height P_H_ (mm)	/	20	20	20	20	20
Pleat width P_W_ (mm)	/	28	14	9.3	7	5.6
Pleat ratio α (P_H_/P_W_)	0	0.71	1.43	2.14	2.86	3.57
Filtration area of the V-shaped filter S_V_ (cm^2^)	143	256	448	652	860	1070
Filtration area of the U-shaped filter S_U_ (cm^2^)	143	327	531	736	940	1145

### 2.2 Experimental system and procedure

**Fig 4:**
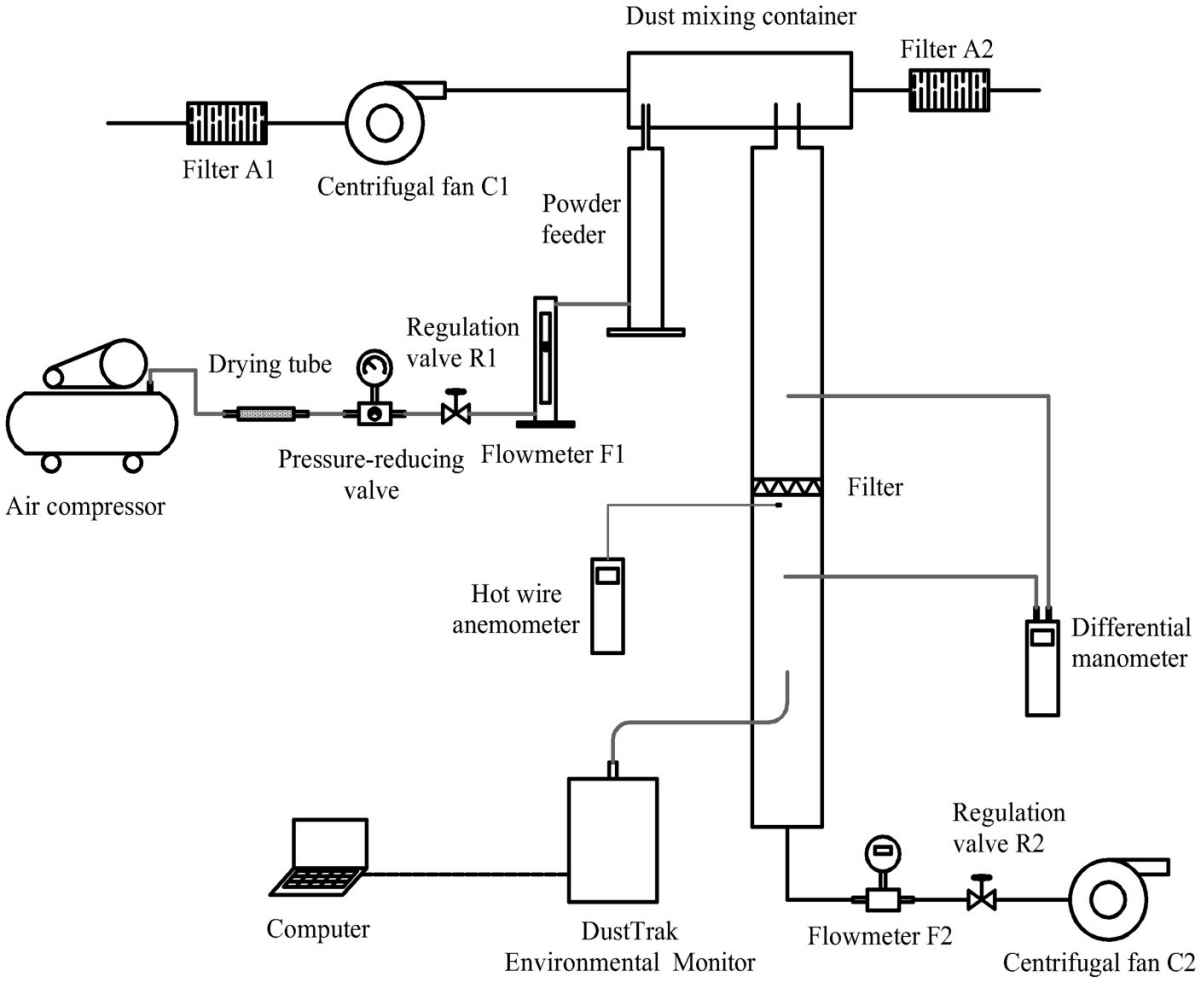
Schematic diagram of the experimental system.

As illustrated in Fig 4, the dust filtration experimental system comprises three parts: the dust generation system, the filtration system and the monitoring system. The dust generation system mainly consists of an air compressor, a drying tube, a pressure-reducing valve, a flow regulation valve R1, a flowmeter F1, a powder feeder (Solid particle generator 9309, TSI, the USA), a dust mixing container and a centrifugal fan C1. The experimental procedure in this part is described as follows: First, high-pressure gas from the air compressor was adjusted to the gas with constant low pressure through the drying tube and the pressure-reducing valve. Subsequently, the gas flow was adjusted through the flow regulation valve R1 so as to control the supply of the powder feeder. Next, the centrifugal fan C1 provided constant airflow to dilute the dust in the dust mixing container and keep its concentration constant. The filtration system included an air filter, a filter tube, a flow regulation valve R2, a centrifugal fan C2 and a flowmeter F2. Among them, the filter tube was composed of two sections of pipes with a length of 400 mm, an outer diameter of 150 mm and an inner diameter of 140 mm. The two sections of pipe clamped the filter through the connecting device. The monitoring system was composed of a differential manometer (AP800, TSI, the USA), a DustTrak environmental monitor (8543, TSI, the USA) and a HWA (VelociCalc air velocity meter Model 9565, TSI, the USA), which were used to measure the pressure drop of the filter, the dust concentration and local air velocity, respectively. For the AP800, the maximum range was 15 inH_2_O; the resolution was 0.001 inH_2_O; and the error was less than 1%. The DustTrak environmental monitor 8543 was employed to monitor the PM_1.0_, PM_2.5_ and total dust concentrations. For the air velocity meter Model 9565, the range was 0–50 m/s; the resolution was 0.01 m/s; and the error was less than 3%.

**Fig 5:**
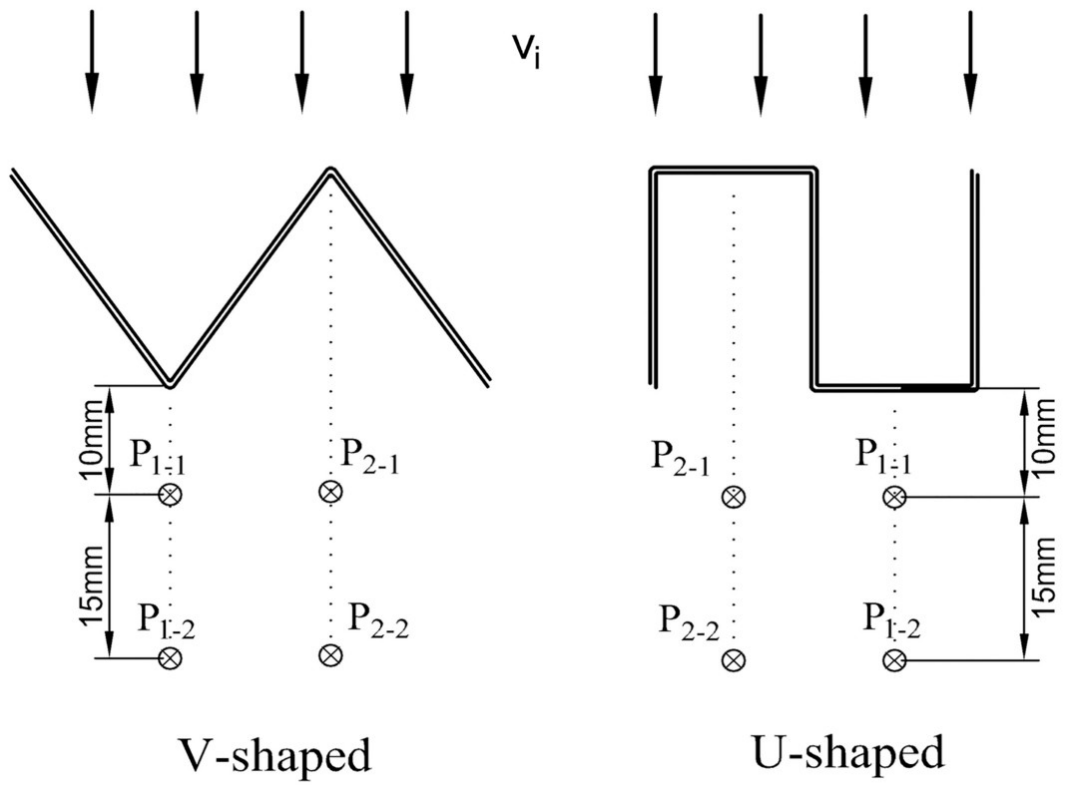
Positions of local air velocity measuring points.

The experiments in this paper were carried out in a dust-free environment and a dust-containing environment, respectively. In the clean airflow, the changes in the pressure drop of the filter with flow rate under different α values were recorded by adjusting the flow regulation valve R2. To verify the influence of the pleated geometry on the local air velocity after the filtration, the filtration velocity was set as 4 cm/s and the flow was altered through the flow regulation valve R2. The specific flow rate data are listed in Table 2. The 9565 HWA was used at 10 mm and 25 mm behind the filter to measure the air velocity, and the specific measuring positions of the probes are shown in Fig 5. The four measuring points were located directly below the pleat corner of the V-shaped filter and the center points of the top and bottom filter surfaces of the U-shaped filter, respectively. To minimize the influence of the pipe wall on the air velocity, the measuring point should be near the center of the filter. The anemometer probe was held parallel to the pleat line for 30 s, and the air velocity was recorded when it stabilized. The experiment was repeated 3 times to ensure reproducibility.

**Table 2:** Flow rate of filters under a filtration velocity of 4 cm/s.

Filter	α = 0	α = 0.71	α = 1.43	α = 2.14	α = 2.86	α = 3.57
Flow rate for the V-shaped filter (L/min)	36.93	64.42	118.84	162.59	214.32	266.47
Flow rate for the U-shaped filter (L/min)	36.93	84.40	137.16	189.91	242.66	295.41

In the dust-containing environment, the dust concentration in the dust-mixing container was maintained at 800±50 mg/m^3^ by adjusting the supply of dust through controlling the powder feeder. The experimental procedure in this part is as follows: First, the regulation valve R2 was adjusted to change the filtration flow rate so that the filtration velocity was maintained at 4 cm/s; afterwards, the filtration duration was controlled until the mass of dust deposition per unit area (*W*) reached 5, 10, 15, 20 and 25 mg/cm^2^, and the number on the micromanometer was recorded; next, the filter was weighed before and after each experiment, and the actual mass of dust deposition per unit area was calculated. The error must be kept within 5%, or the experiment needed to be restarted. The above experiment was repeated 3 times to ensure reproducibility.

### 2.3 Numerical simulations

**Fig 6:**
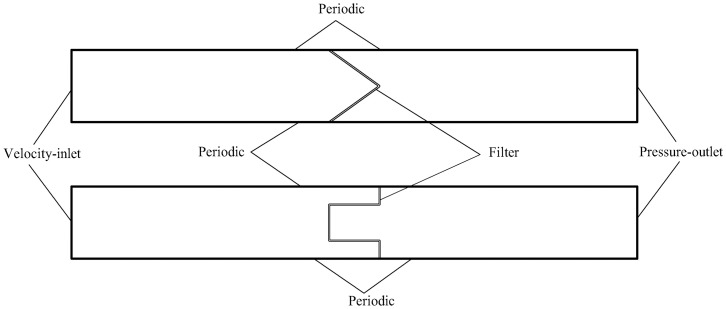
Numerical simulation computational zone.

To simulate the velocity field of pleated filter, a two-dimensional computational domain is hereby established. Its computational zone and boundary conditions are depicted in Fig 6. The computational zone is divided into three parts, i.e., the pleated filter media zone, the upstream velocity field zone of the filter and the downstream velocity field zone of the filter. Among them, the pleated filter media zone is an isotropic porous media area where the performance variation of the bending zone of the filter media can be ignored. The upstream and downstream velocity field zones are both 100 mm long. Their left and right sides are the velocity-inlet and the pressure-outlet, respectively. The upper and lower sides are periodic boundaries that serve to reduce the influence of the boundary layer on the velocity field. The computational zone is subject to unstructured meshing, in which the mesh sizes of the porous media zone and other zones are 0.05 mm and 0.2 mm, respectively. The computational domains of the V-shaped and U-shaped filters under different α values are meshed. Upon examination, the pressure drop and velocity distribution in finer meshes are basically the same with values obtained here, so the meshing in this study is reasonable.

When the filtration velocity is 4 cm/s, the inlet air velocities of the V-shaped and U-shaped filters under different α values are described in Table 3. According to the calculation results, the Reynolds number (Re) ranges from 669 to 3,122. In this paper, the Lam and k-ω (ST), k-ω (LR-SST), k-ε (LR) and v2f turbulent models in ANSYS Fluent 19.0 software were used to explore the pressure drop and the velocity field distribution of the V-shaped and U-shaped filters with varying α values. The characteristics of turbulent models are shown in Table 4. In the numerical simulation, the air density was set to 1.2 kg/m^3^, the dynamic viscosity to 1.8156×10^−5^ Pa·s, the gauge pressure to 0 Pa at the outlet, and residuals to not greater than 10^−3^. Besides, pressure-velocity coupling was performed using the SIMPLEC algorithm.

**Table 3:** Inlet air velocity under a filtration velocity of 4 cm/s.

Filter	α = 0.71	α = 1.43	α = 2.14	α = 2.86	α = 3.57
Inlet air velocity for the V-shaped filter v_i_ (cm/s)	6.979	12.114	17.612	23.216	28.865
Inlet air velocity for the U-shaped filter v_i_ (cm/s)	9.714	15.429	21.143	26.857	32.571

**Table 4:** Characteristics of turbulent models.

Turbulent model	Characteristic
k-ω (ST)	K and ω are solved on the basis of the model of two transfer equations. The model boasts a better flow effect in the presence of a wall and under a low Re [16, 24, 25].
k-ω (LR-SST)	k-ω (ST) model is modified to adapt to the zone with a small Reynolds number, thus forming the k-ω (LR-SST) turbulent model [17].
k-ε (LR)	The standard k-ε model is only suitable for the complete turbulent flow. In the case of a small Reynolds number, it needs to be modified into the k-ε (LR) turbulent model [26].
v2f	V2f is a model between the turbulence viscosity coefficient model and the Reynolds stress model. It is a general turbulent model with a low Reynolds number [16, 27].

During dust loading, the pleated structure of the filter affects the trajectory of the particles [3, 18, 19, 21, 28–30] and leads to the uneven deposition of dust particles [6, 11, 31]. Some scholars found that the dust deposition on the pleated filter media is closely related to the Stokes number (*S*_*tk*_), which can be expressed as Eq (1) [18, 30].

$$S_{tk}=\frac{\rho_{p}d_{p}^{2}v_{i}}{18\mu L_{0}}$$ (1)

where *ρ*_*p*_ is the particle density (kg/m^3^), *d*_*p*_ is the particle diameter (m), *v*_*i*_ is the inlet air velocity (m/s), *L*_0_ is the characteristic length of the obstacle (m), *L*_0_ takes values *P*_*W*_ and 0.5*P*_*W*_ for V-shaped and U-shaped filters, respectively.

Saleh [18] reported that when *S*_*tk*_ < 0.1, particles are evenly distributed in the airflow and can move closely with the streamline without being affected by obstacles. Bourrous [21] found that when *S*_*tk*_ ≪ 1, the inertia of the particles at the entrance to the pleat is negligible. The average diameter of the dust particles in this study is 2.39 μm, and 99% of the particles are smaller than 10 μm. At a filtration velocity of 4 cm/s, the *S*_*tk*_ for the 10 μm particles in the experiment calculated by Eq (1) is only 0.07. Therefore, it is reasonable to ignore the inertia of the particles and consider that the dust cake thickness is proportional to the NAVF. Numerical simulations can be used to derive the NAVF and thus simulate the change in dust cake thickness during dust loading.

## 3 Results and discussion

### 3.1 Influence of pleated geometry on pressure drop

The pressure drop of the pleated filter has been investigated in many previous studies [28, 32–35]. Some scholars divided the total pressure drop into the pressure drop of the pleated geometry and that of the filter media [34, 35], while others ignored the former considering that the structural resistance is much smaller than the filtration resistance under a low filtration velocity [28, 32, 33]. In this paper, the structural resistance was neglectable, and the pressure drop of the filter media was regarded as the total pressure drop which can be calculated by the Darcy’s law [28, 32, 33].

$$\Delta P_{T}=\frac{\mu D_{F}}{K_{F}}\nu_{f}=k\nu_{f}$$ (2)

where Δ*P*_*T*_ is the pressure drop (Pa), *μ* is the dynamic viscosity of air (Pa·s), *D*_*F*_ is the thickness of the filter media (m), *K*_*F*_ is the permeability coefficient of the filter media (m^2^), *v*_*f*_ is the filtration velocity (m/s), and *k* is the resistance coefficient (Pa·s/cm).

**Fig 7:**
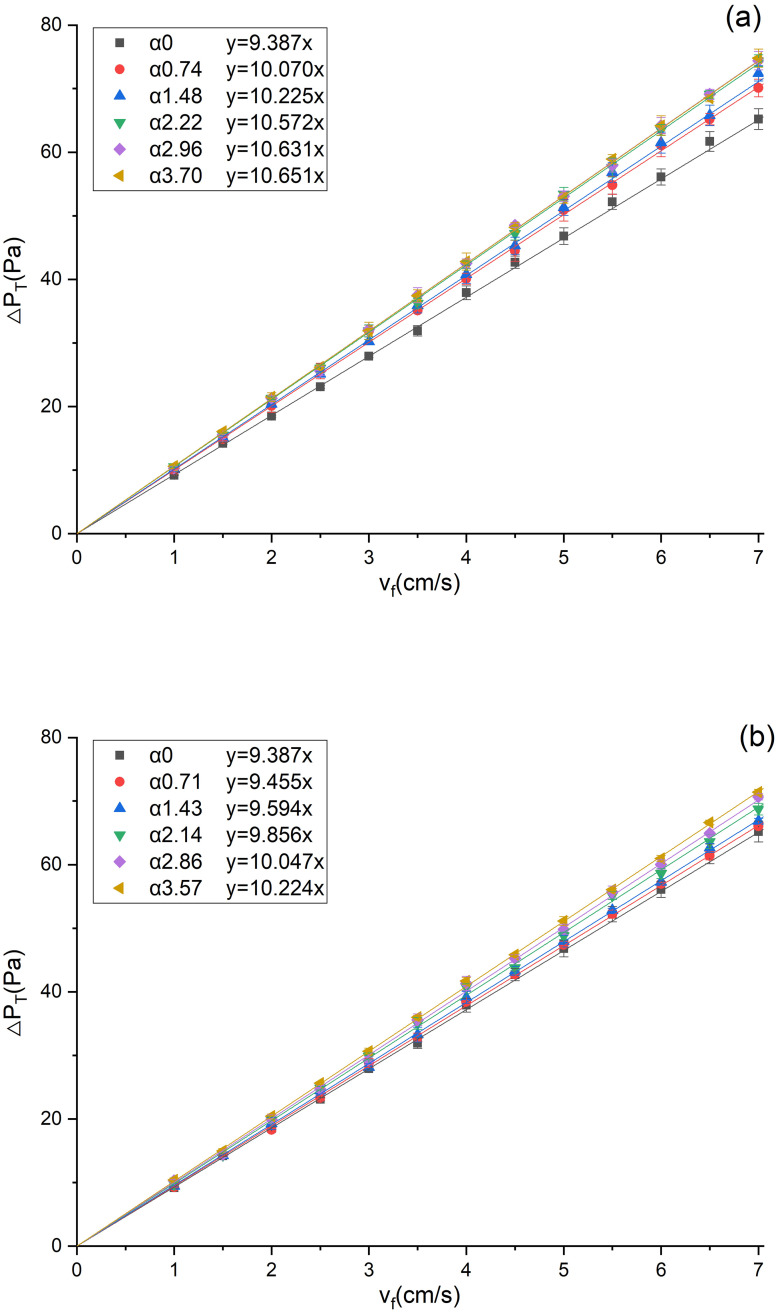
Variations of the total pressure drop with filtration velocity (a)V-shaped, (b) U-shaped.

The total pressure drops under different α values were measured in a dust-free environment, as shown in Fig 7. It can be found that the total pressure drop of the filter increases linearly with the filtration velocity, and the increase is faster under a greater α value. This phenomenon is caused by the changes in parameters such as the thickness, porosity and permeability of the bending zone of the filter media [7, 14, 36]. Linear fitting of the data by Eq (2) found that the fitting coefficients are greater than 0.99, indicating that it was reasonable to ignore the structural resistance of the pleated filter. The resistance coefficients of the V-shaped and U-shaped filters, which equal the slopes of the corresponding fitting lines, are 9.387–10.651 Pa·s/cm and 9.387–10.224 Pa·s/cm, respectively. It can be seen that the U-shape filter has a smaller resistance coefficient under the same α, indicating that its pleated geometry has a slighter influence on the total pressure drop.

### 3.2 Selection of numerical model

**Fig 8:**
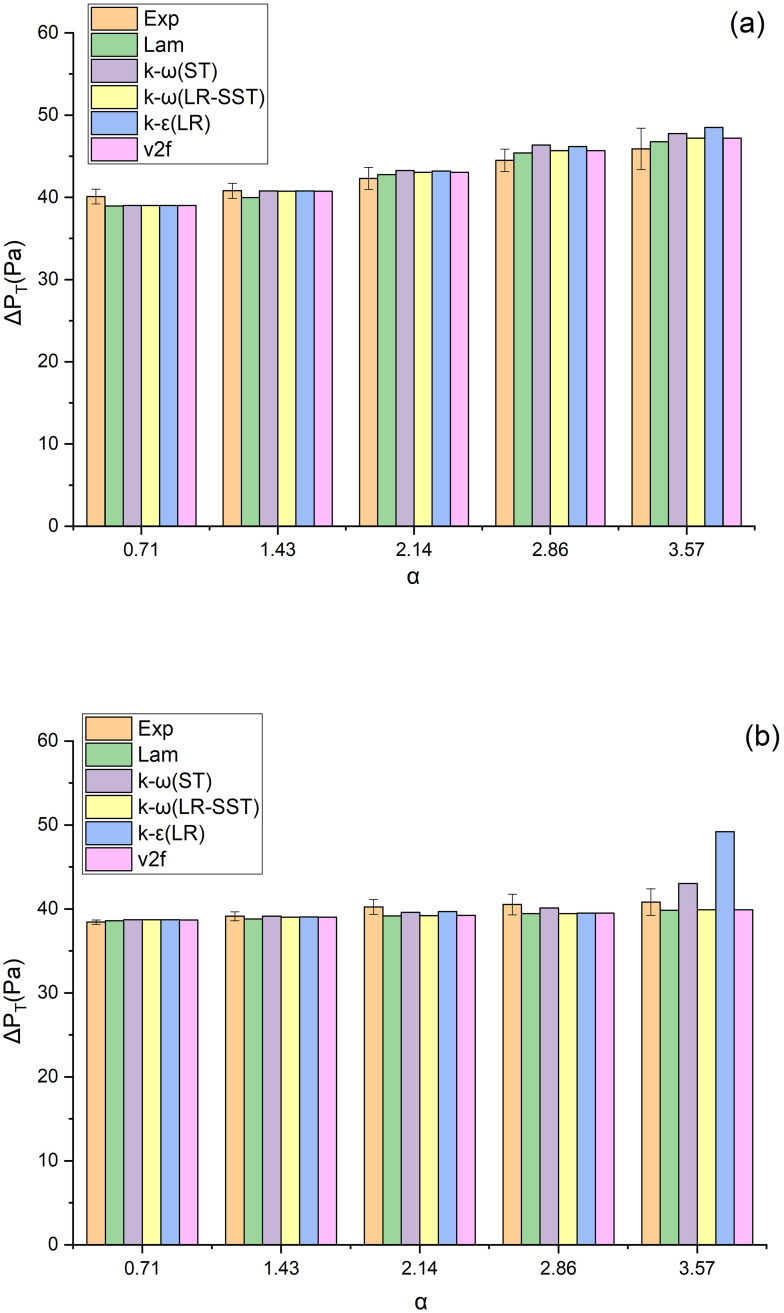
Pressure drops of the filter under different CFD models (a) V-shaped, (b) U-shaped.

When the filtration velocity is 4 cm/s, the Lam, k-ω (S), k-ω (LR-SST), k-ε (LR) and v2f models were adopted for numerical simulation on the pleated filter, and the obtained pressure drops were compared with the experimentally measured values (Fig 8). It is found that except for the k-ε (LR) model of the U-shaped filter under the α value of 3.57, the errors between the simulated pressure drops and the experimental ones are all within 10% in other models. This indicates that the models can predict the pressure drop well.

**Fig 9:**
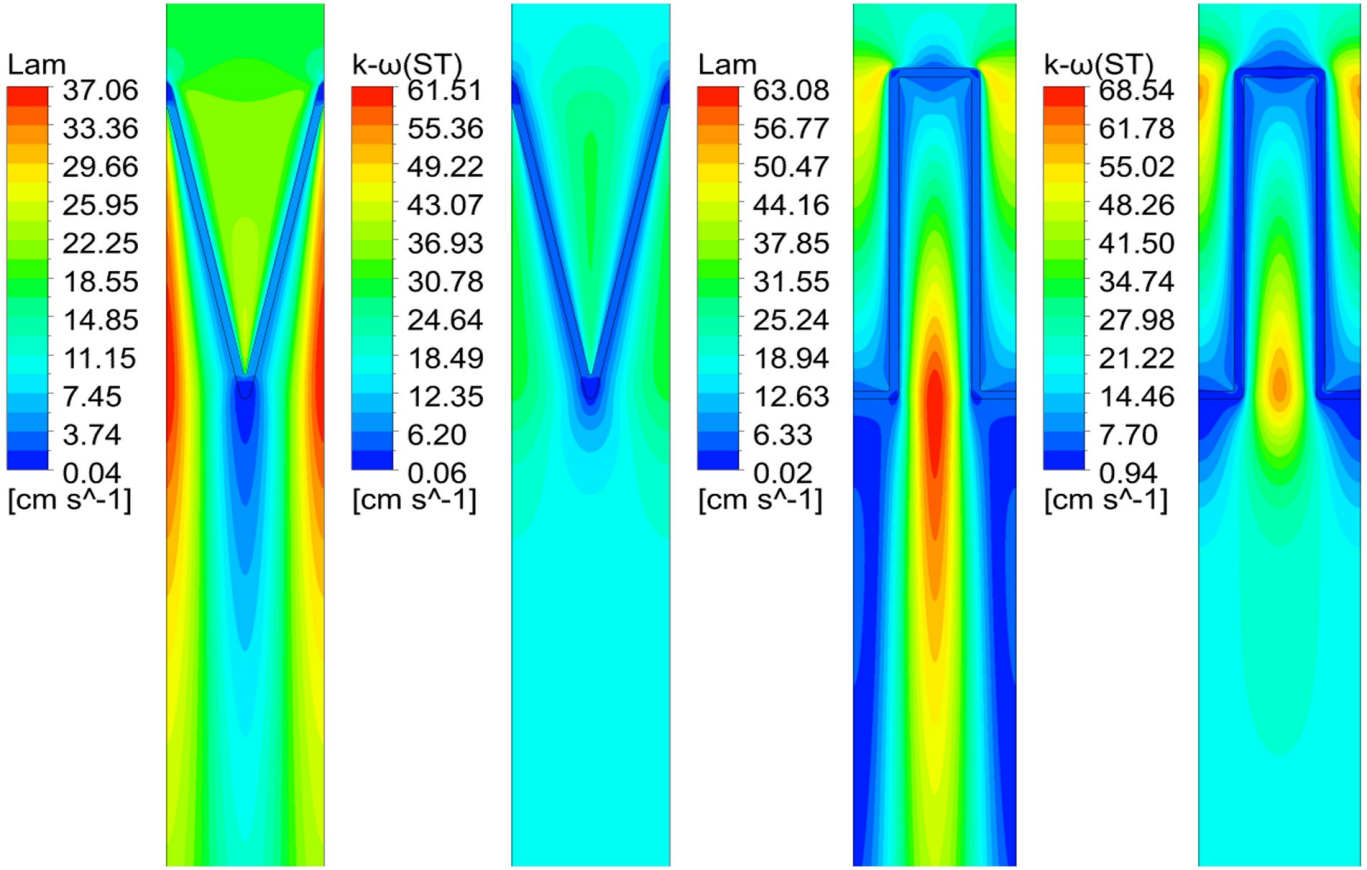
Velocity fields under different CFD models when α = 2.14.

However, the numerical simulation demonstrates that the velocity fields of the V-shaped and U-shaped filters differ significantly under different CFD models, which is attributed to the different conditions applicable to each model. For the sake of a more straightforward comparison of velocity fields under different CFD models, this study selects the velocity fields under the Lam and k-ω (ST) models when α = 2.14 as an example for analysis (Fig 9). It can be seen that the velocity fields under different CFD models differ greatly, mainly in the post-filtration air velocity distribution among pleats. These differences indicate that it is unreliable to determine the applicability of the model merely by pressure drop. Instead, it needs further verification based on the velocity field.

**Fig 10:**
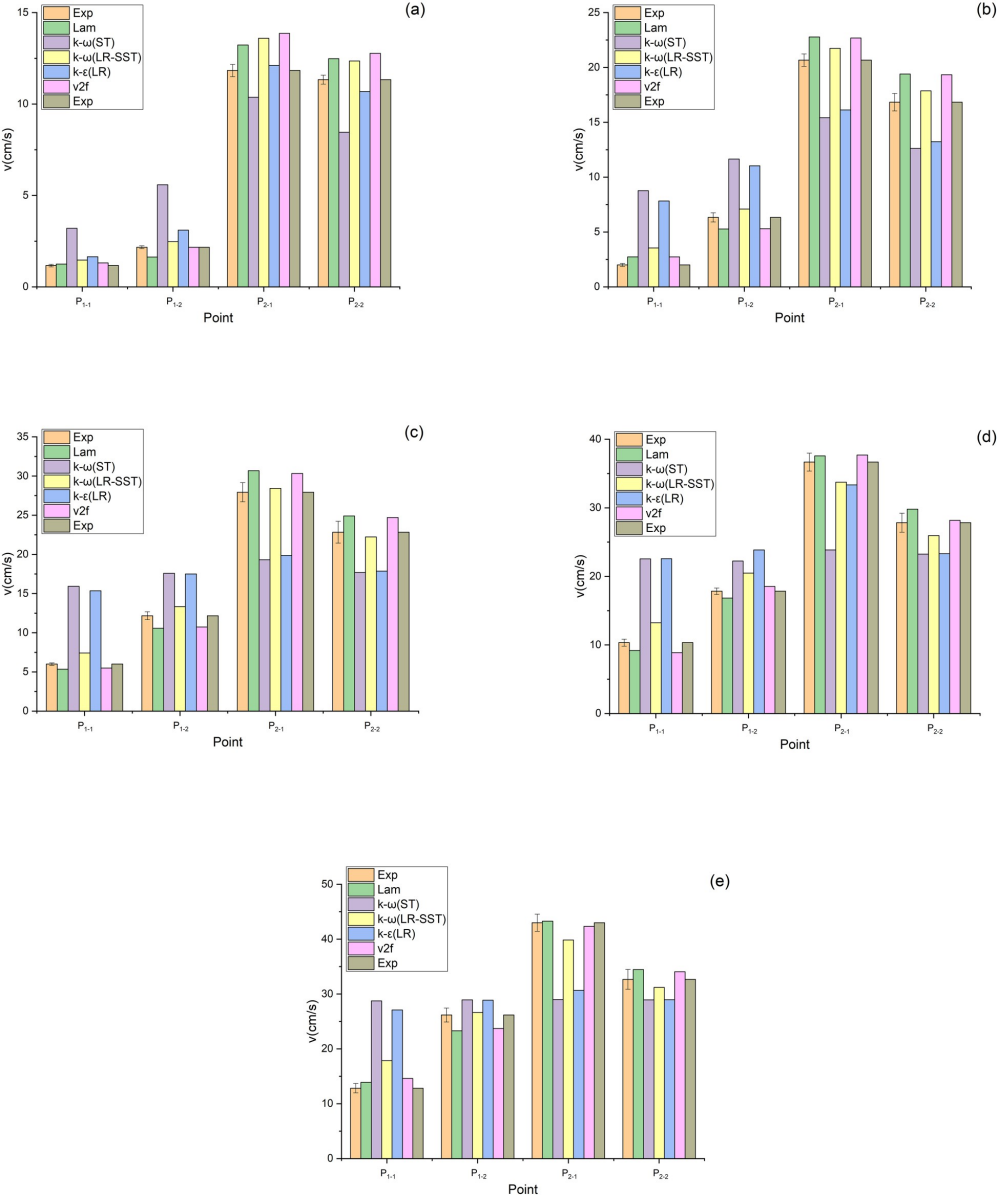
Air velocities of the V-shaped filters at measuring points under different CFD models (a) α = 0.71, (b) α = 1.43, (c) α = 2.14, (d) α = 2.86, (e) α = 3.57.

**Fig 11:**
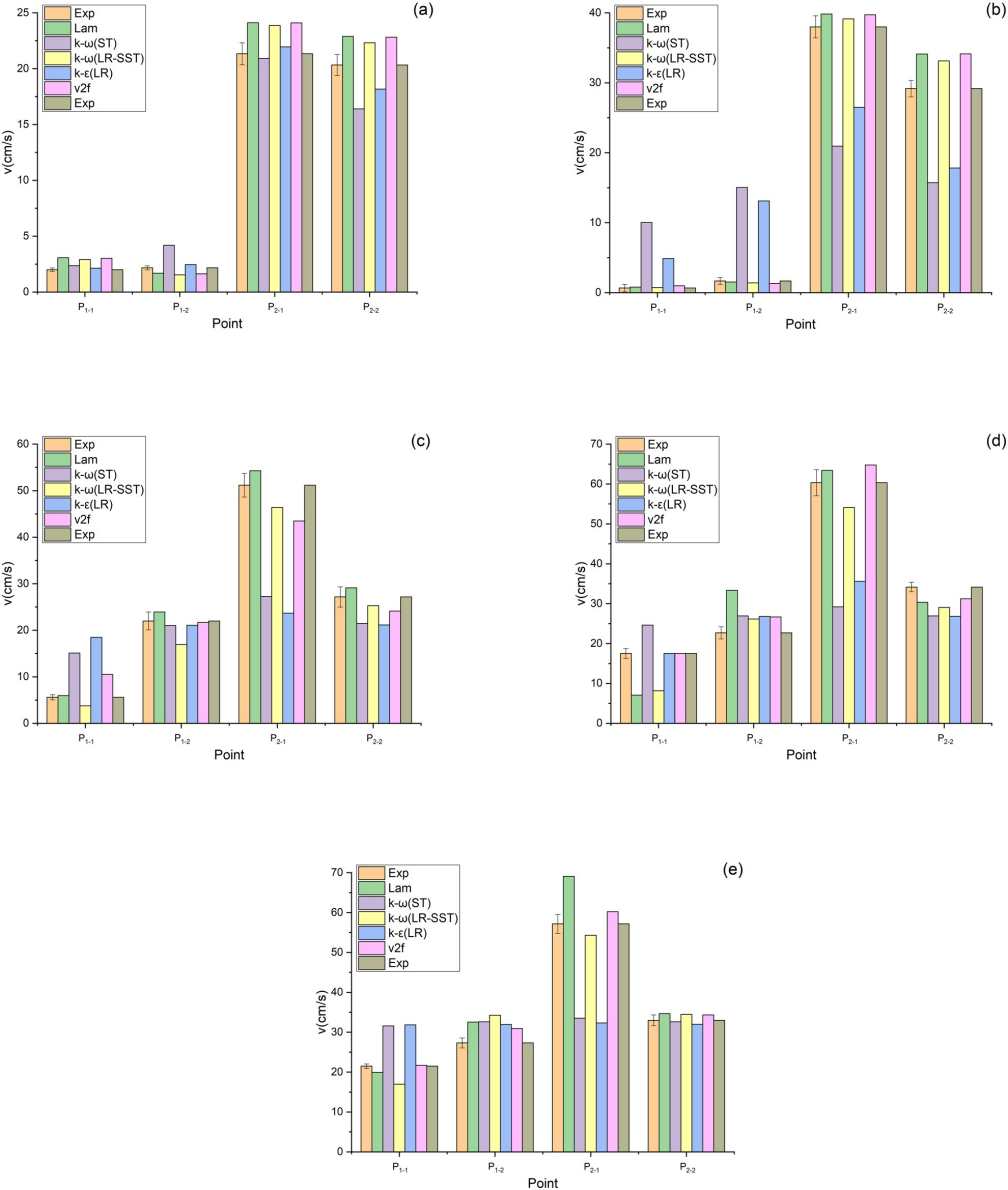
Air velocities of the U-shaped filters at measuring points under different CFD models (a) α = 0.71, (b) α = 1.43, (c) α = 2.14, (d) α = 2.86, (e) α = 3.57.

The air velocities at the measuring points shown in Fig 5 were measured with the aid of a HWA, as shown in Figs 10 and 11. Fig 10 shows the air velocities at the measuring points of the V-shaped filters under different α values. As exhibited in Fig 10(a), when α equals 0.71, the data obtained by the Lam, k-ω (LR-SST), k-ε (LR) and v2f models agree with the experimental data. As presented in Fig 10b–10e, when α equals 1.43–3.57, the data obtained by the Lam, k-ω (LR-SST) and v2f models are close to the experimental data. Therefore, the Lam, k-ω (LR-SST) and v2f models are applicable to the V-shaped filters.

Fig 11 shows the air velocities at the measuring points of the U-shaped filters under different α values. As illustrated in Fig 11(d), when α equals 2.86, only the data obtained by the v2f model are in agreement with the experimental data. Fig 11(e) demonstrated that the v2f model can also predict the air velocity when α equals 3.57. As indicated in Fig 11a–11c, when α equals 0.71–2.14, the data obtained from the Lam and k-ω (LR-SST) models are consistent with the experimental data. Therefore, for the U-shaped filter, the Lam and k-ω (LR-SST) models can be used when α equals 0.71–2.14, and the v2f model can be used when α equals 2.86–3.57. The Lam model is inapplicable when α is large, which may be caused by the fact that the gas flow state is no longer laminar.

### 3.3 Numerical simulation of dust loading

**Fig 12:**
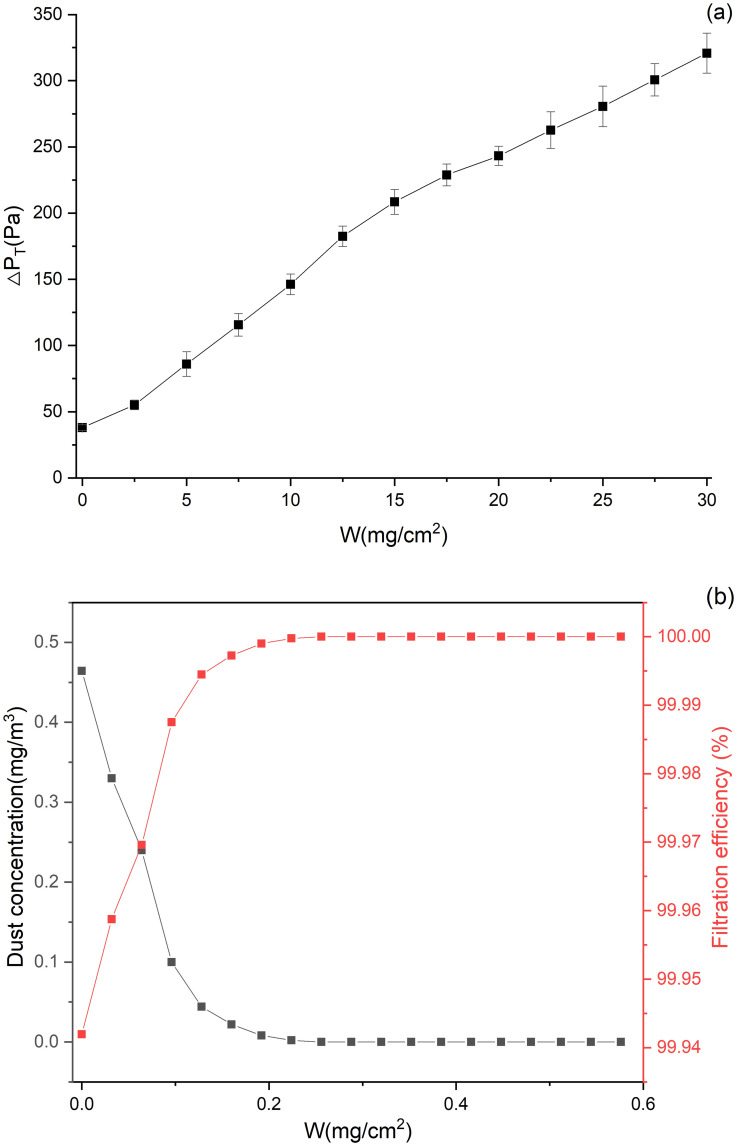
Variation of pressure drop and filtration efficiency of flat filtration during dust loading.

Fig 12 depicts the variation of pressure drop and filtration efficiency of flat filtration during dust loading. In the experiment, the dust concentration and the filtration velocity were set as 800 mg/m^3^ and 4 cm/s, respectively. As revealed by Fig 12(a), the total pressure drop increases as *W* rises; besides, the rate of increase first climbs and subsequently drops, due to the change in the dust deposition characteristics. Some scholars [33, 37, 38] found that the variation of pressure drop with dust deposition falls into 3 stages: the deep filtration stage, the transition stage and the surface filtration stage. Specifically, deep filtration occurs in the initial stage of filtration. At this stage, large particles deposit on the surface of the filter media while fine particles enter the filter media and get trapped, resulting in smaller pores of filter media and improved filtration efficiency. According to Fig 12(b), the filtration efficiency rapidly climbs from 99.9% to 100% during dust loading. Then, it enters the transition stage, in which a dust cake is formed on the surface of the filter media, indicating that deep filtration is substituted by surface filtration. The third stage is dominated by surface filtration, where the pressure drop increases linearly and the filtration efficiency hits its maximum. As demonstrated in Fig 12(a), when W>15 mg/cm^2^, the total pressure drop rises approximately linearly, which is a mark of surface filtration; in contrast, when W<15mg/cm^2^, it is in the deep filtration and transition stages. In this study, dust is assumed to merely deposit on the surface of the filter media. Under such an assumption, the permeability coefficients of dust cake under different *W* values during flat filtration can be calculated.

For flat filtration, the total pressure drop, which consists of the pressure drops caused by both the filter media and the dust cake, can be expressed as Eq (3) according to Darcy’s law [28, 32]:

$$\Delta P_{T}=\Delta P_{F}+\Delta P_{C}=\Delta P_{F}+\frac{\mu W\nu_{f}}{K_{C}\rho }$$ (3)

where Δ*P*_*T*_ is the total pressure drop (Pa), Δ*P*_*F*_ and Δ*P*_*C*_ are the pressure drops caused by the filter media and the dust cake, respectively (Pa), *W* is the mass of dust deposition per unit area (kg/m^2^), *v*_*f*_ is the filtration velocity (0.04 m/s), *K*_*C*_ is the permeability coefficient of dust cake (m^2^), ρ is the bulk density of dust (620 kg/m^3^). Δ*P*_*T*_ and Δ*P*_*F*_ under different *W* values were obtained by experiments. In this study, it is assumed that under the same *W*, the *K*_*C*_ values of pleated filtration and flat filtration are the same. By Eq (3), *K*_*C*_ could be expressed as:

$$K_{C}=\frac{\mu W\nu_{f}}{\left(\Delta P_{T}-\Delta P_{F}\right)\rho }$$ (4)

Based on the conclusion drawn in Section 3.2, the Lam model is used for the V-shaped filter; for the U-shaped filter, the Lam model is employed when α equals 0.71–2.14 and the v2f model is adopted when α equals 2.86–3.57.

**Fig 13:**
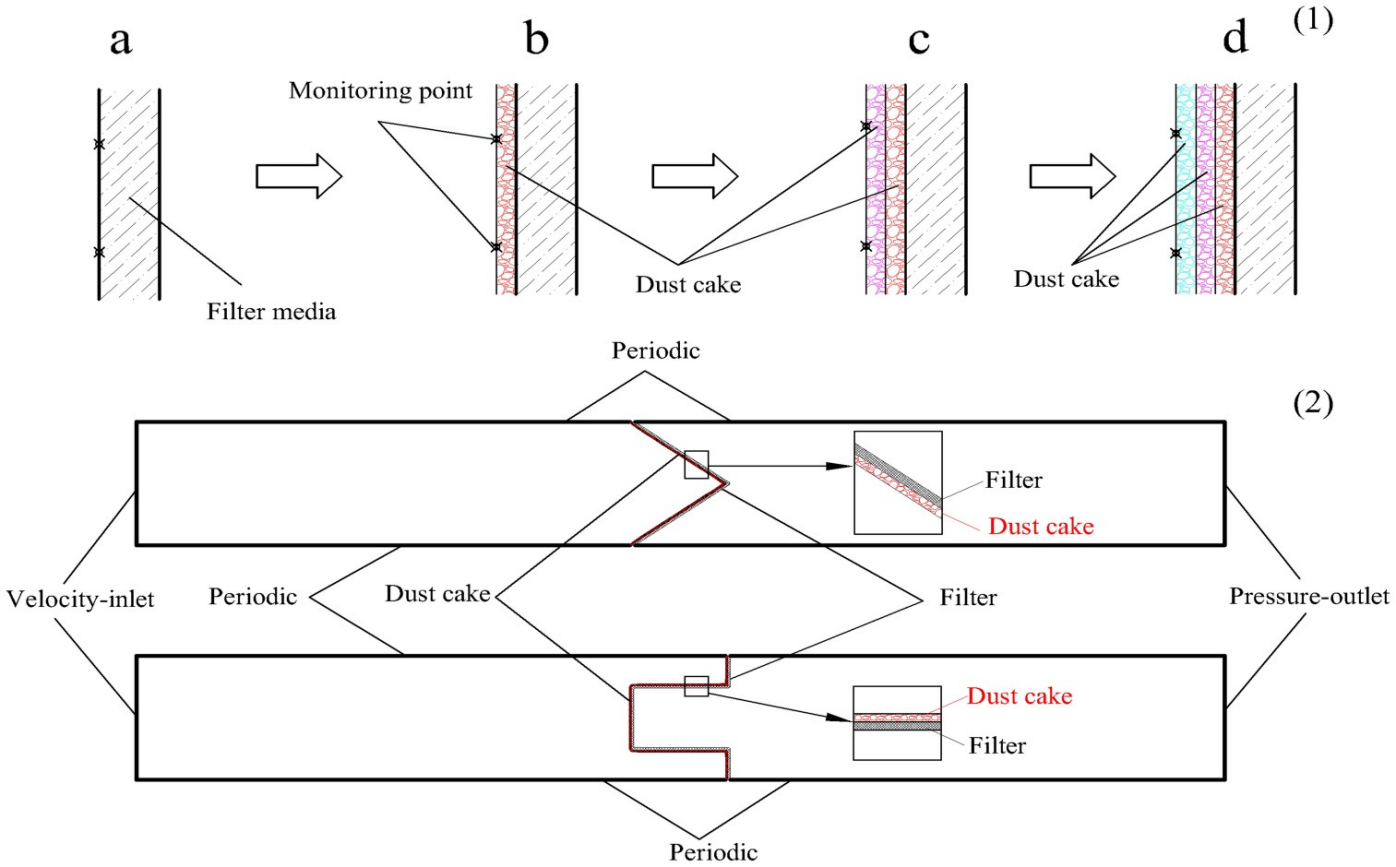
Numerical simulation computational zone during dust loading (1) formation of dust cake during dust loading (2) computational domain.

In this paper, the dust cake thickness is considered to be proportional to the NAVF. Since the pleats are symmetrical, the monitoring points were spaced about 1 mm apart on a half of the pleats, as displayed in Fig 13(1). The thickness of dust cake at each monitoring point could be obtained through Eq (5).

$$D_{C}^{\prime }=\frac{\int_{0}^{t}C\nu_{t}dt}{\rho }$$ (5)

where $D_{C}^{\prime }$ is the dust cake thickness at the monitoring point (m), *C* is the dust concentration (kg/m^3^), and *v*_*t*_ is the NAVF at the monitoring point at time t (m/s). Then, the increase dust cake thickness at the monitoring point from t_1_ to t_2_ can be calculated according to Eq (6).

$$\Delta D_{C}^{\prime }=\frac{C}{\rho }\left(\int_{0}^{t_{2}}\nu_{t}dt-\int_{0}^{t_{1}}\nu_{t}dt\right)$$ (6)

where $\Delta D_{C}^{\prime }$ is the increased dust cake thickness at the monitoring point (m). Eq (6) can also be expressed as:

$$\Delta D_{C}^{\prime }=\frac{\Delta W}{\rho \nu_{f}}{\bar{\nu }}_{t_{1}-t_{2}}$$ (7)

where ${\bar{\nu }}_{t_{1}-t_{2}}$ is the average NAVF at the monitoring point from t_1_ to t_2_ (m/s), Δ*W* is the increased mass of dust deposition per unit area (kg/m^2^), and *v*_*f*_ is the filtration velocity (0.04 m/s). When Δ*W* is extremely small, ${\bar{\nu }}_{t_{1}-t_{2}}$ is considered to be equal to the NAVF at the monitoring point at time t_1_ ($\nu_{t_{1}}$), which can be obtained through numerical simulation. Thus,

$$\Delta D_{C}^{\prime }=\frac{\nu_{t_{1}}\Delta W}{\rho \nu_{f}}$$ (8)

With the U-shaped filter as an example, the formation of dust cake during dust loading is illustrated in Fig 13(1). In dust cake formation on the surface of a clean filter, the NAVF at the monitoring point on the surface of the filter media can be obtained through the numerical simulation described in Section 3.2. When Δ*W* is determined, the increased dust cake thickness at monitoring point on the surface of the filter media can be calculated by Eq (8). Afterwards, the dust cake can be determined by connecting the measuring points, as displayed in Fig 13(1b). This method can also be used to increase the thickness of the dust cake. The NAVF at the monitoring point on the surface of the dust cake can be obtained by numerical simulation, and then the increased dust cake thickness at monitoring point can be calculated by Eq (8), as displayed in Fig 13c and 13d.

The method of numerical simulation of filters with dust cake is described below. According to Fig 13(2), the computational domain is divided into four zones, i.e., the upstream velocity field zone of the filter, the dust cake zone, the filter zone and the downstream velocity field zone of the filter media. Next, the unstructured meshing described in Section 2.3 and the numerical simulation are performed on the computational domain, where the dust cake zone is a porous media area with a mesh size of 0.02 mm. The permeability coefficient of the dust cake can be calculated by Eq (3). Thus, the NAVF at the monitoring point on the surface of the dust cake can be obtained by numerical simulation. In the numerical simulations, the smaller Δ*W* is, the smaller the error is, but the greater the workload. In this paper, Δ*W* is taken as 10 mg/cm^2^, 5 mg/cm^2^ and 2.5 mg/cm^2^, respectively.

**Fig 14:**
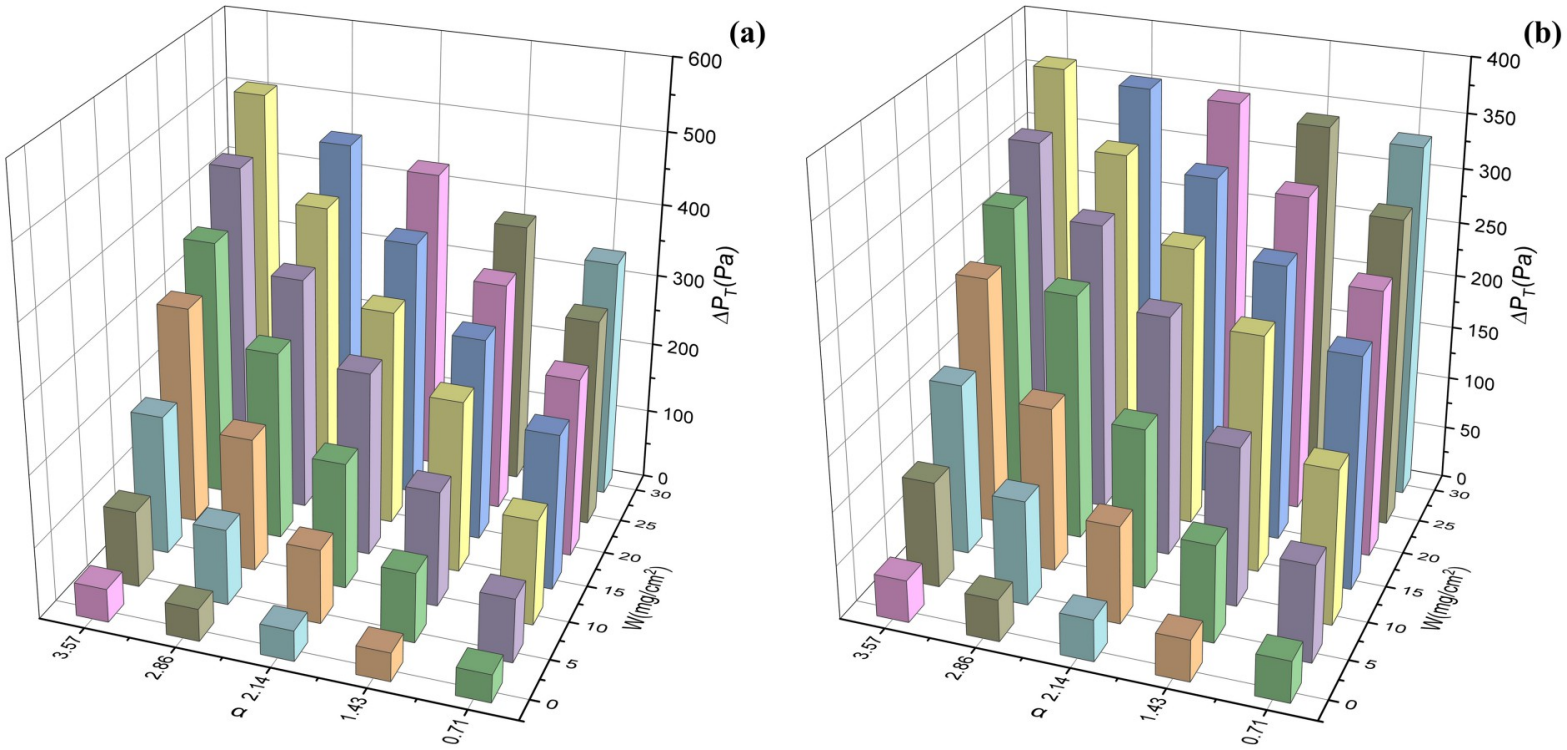
Changes in simulated total pressure drops with α and *W* of the filters (a) V-shaped filters, (b) U-shaped filters.

In the numerical simulation, it is found that the total pressure drop calculated under the Δ*W* of 10 mg/cm^2^ differs greatly from that measured by the experiment, while the errors are within 7% under the Δ*W* of 5 mg/cm^2^ and 2.5 mg/cm^2^. Therefore, the value of Δ*W* is set as 5 mg/cm^2^ to reduce the workload of numerical simulations. Fig 14 present the changes in simulated total pressure drops with α and *W* of V-shaped and U-shaped filters. The experimental pressure drop was measured by the experimental system in Fig 4. The relative errors of experimental and simulated total pressure drops range from -4.62% to 6.48% and from -2.48% to 3.34%, and their relative mean deviations are 3.12% and 1.19%, respectively. The pressure drop during dust loading can be accurately predicted by numerical simulation. As illustrated in Fig 14, the pressure drops of the V-shaped and U-shaped filters increased with the growth of *W* and α. Moreover, under the same α and *W*, the total pressure drop of the U-shaped filter was lower than the V-shaped filter.

### 3.4 Influence of dust deposition on the unevenness of the NAVF

**Fig 15:**
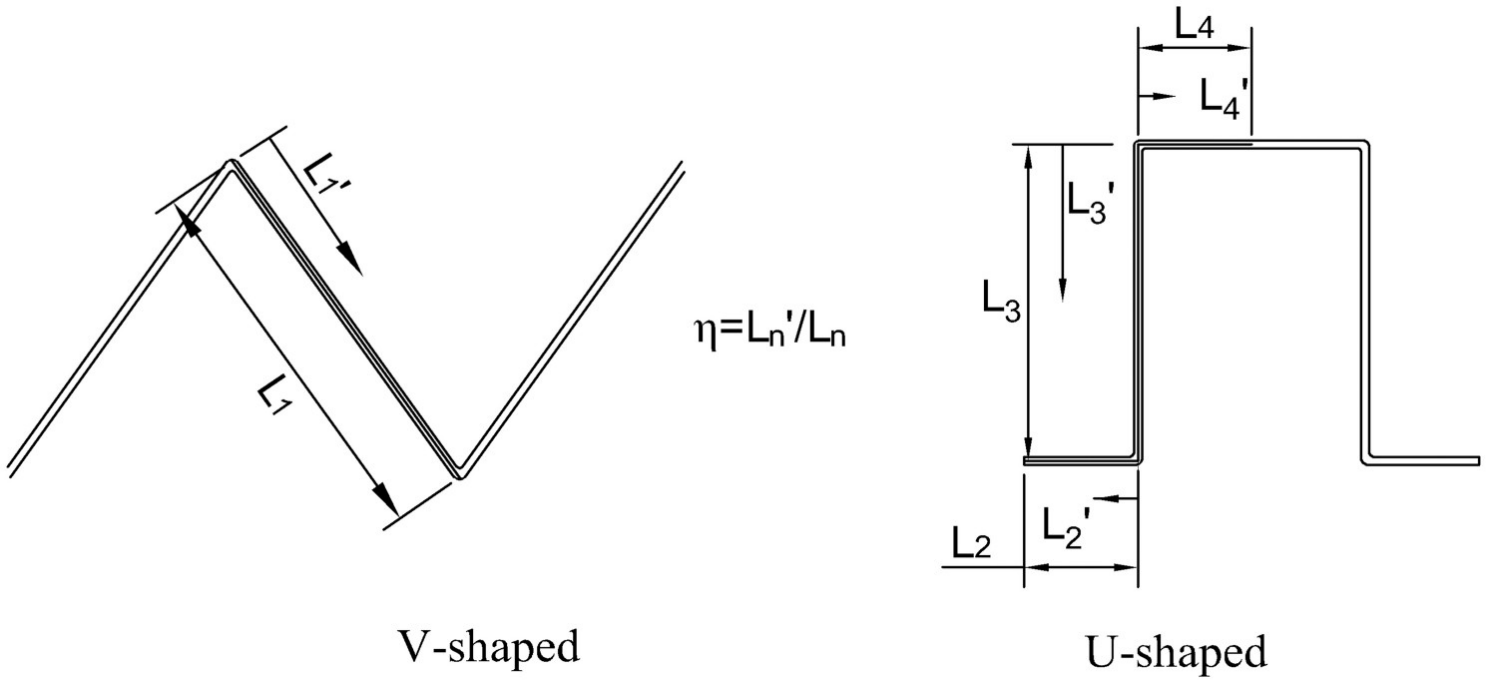
Positions of the NAVF monitoring points and dimensionless constant η (η represents the position of the monitoring point; η equals 0 at the starting point and 1 at the end point).

The pleated geometry and the uneven dust deposition can lead to variations of the NAVF. In this study, the NAVF is defined as the normal air velocity at the centerline of the filter. As for the measurement of the NAVF, 5 monitoring points are spaced per mm at the centerline of the filter media (Fig 15).

**Fig 16:**
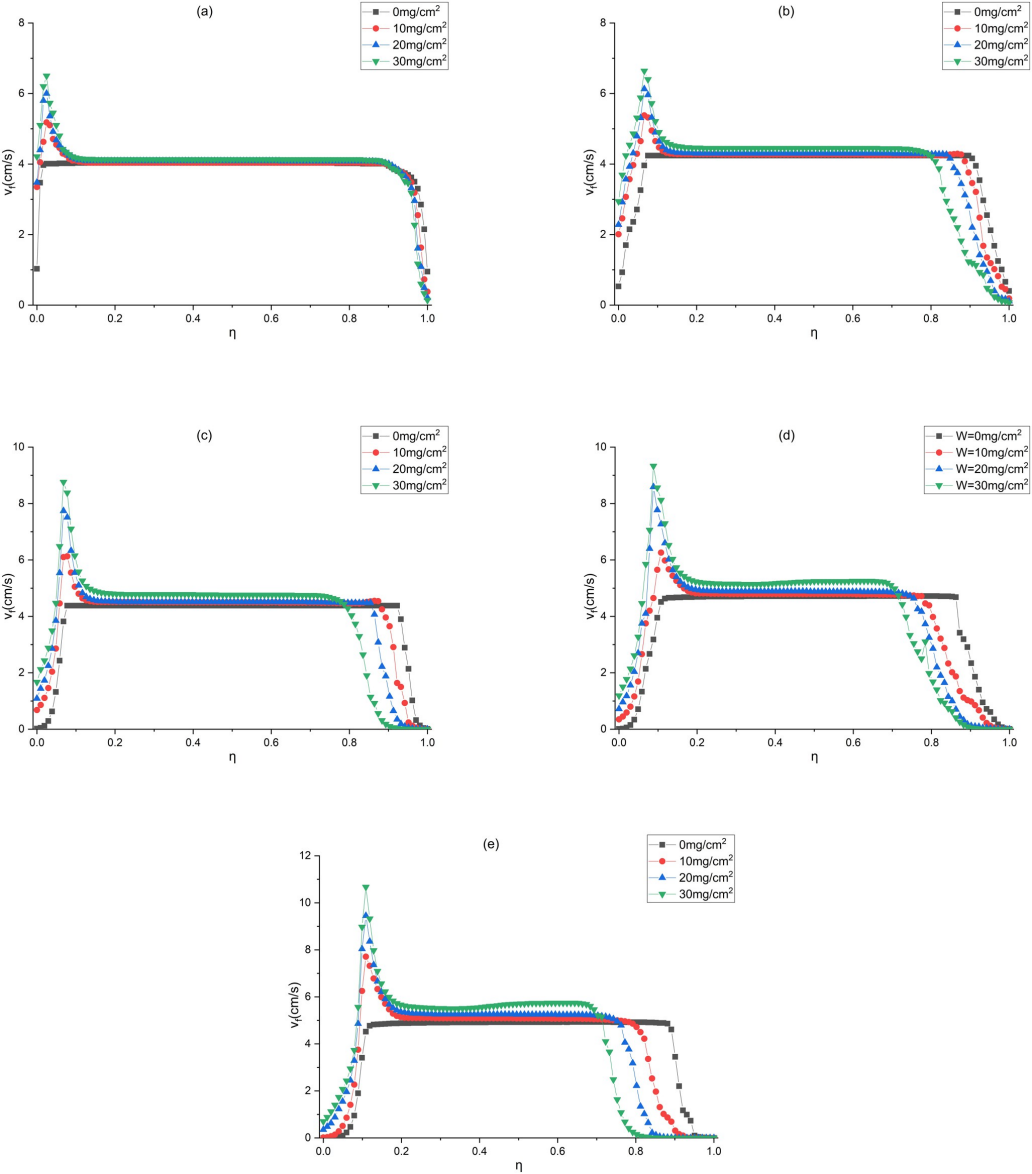
Changes in the NAVFs of the V-shaped filter during dust loading (a) α = 0.71, (b) α = 1.43, (c) α = 2.14, (d) α = 2.86, (e) α = 3.57.

**Fig 17:**
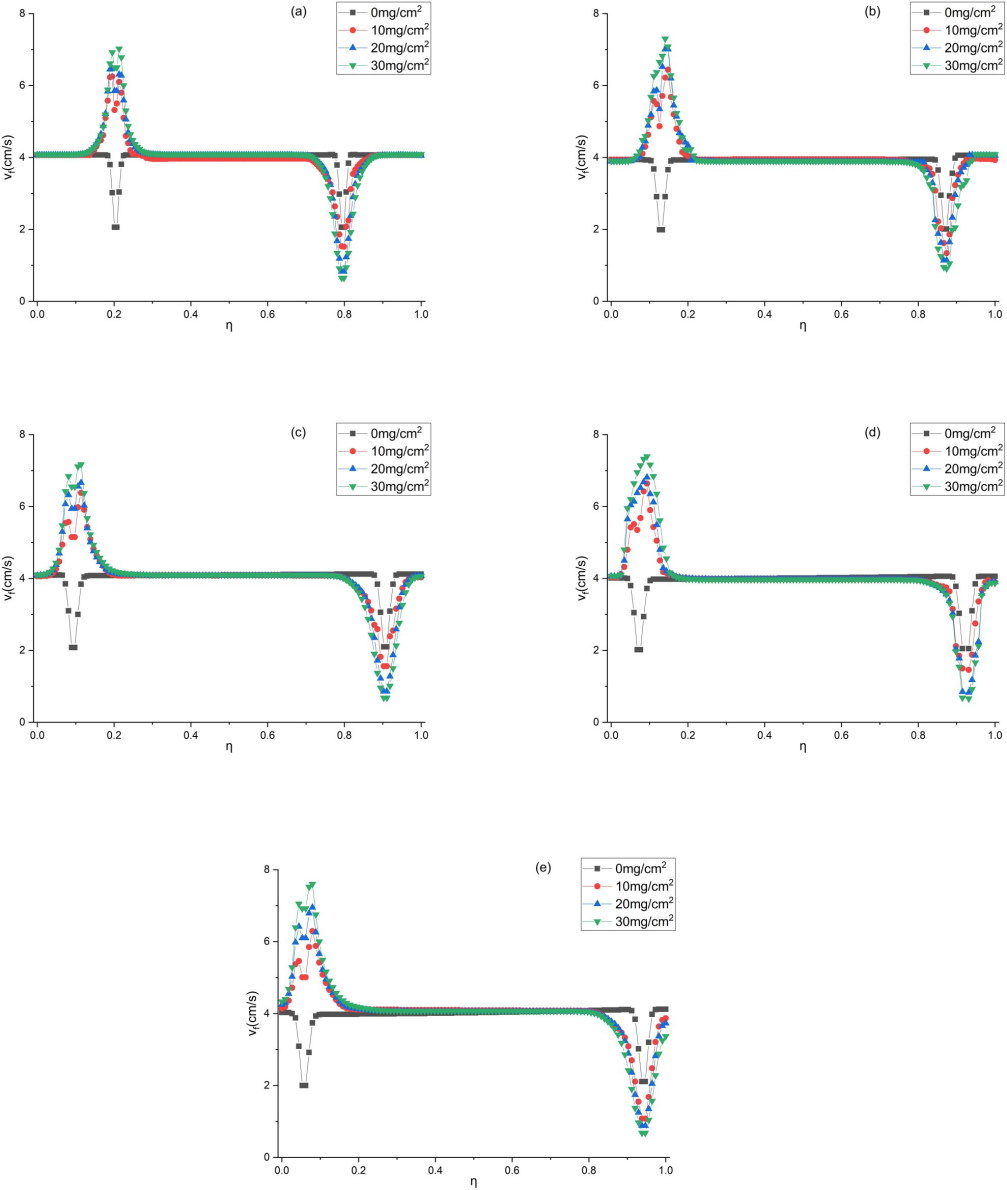
Changes in the NAVFs of the U-shaped filter during dust loading (a) α = 0.71, (b) α = 1.43, (c) α = 2.14, (d) α = 2.86, (e) α = 3.57.

**Fig 18:**
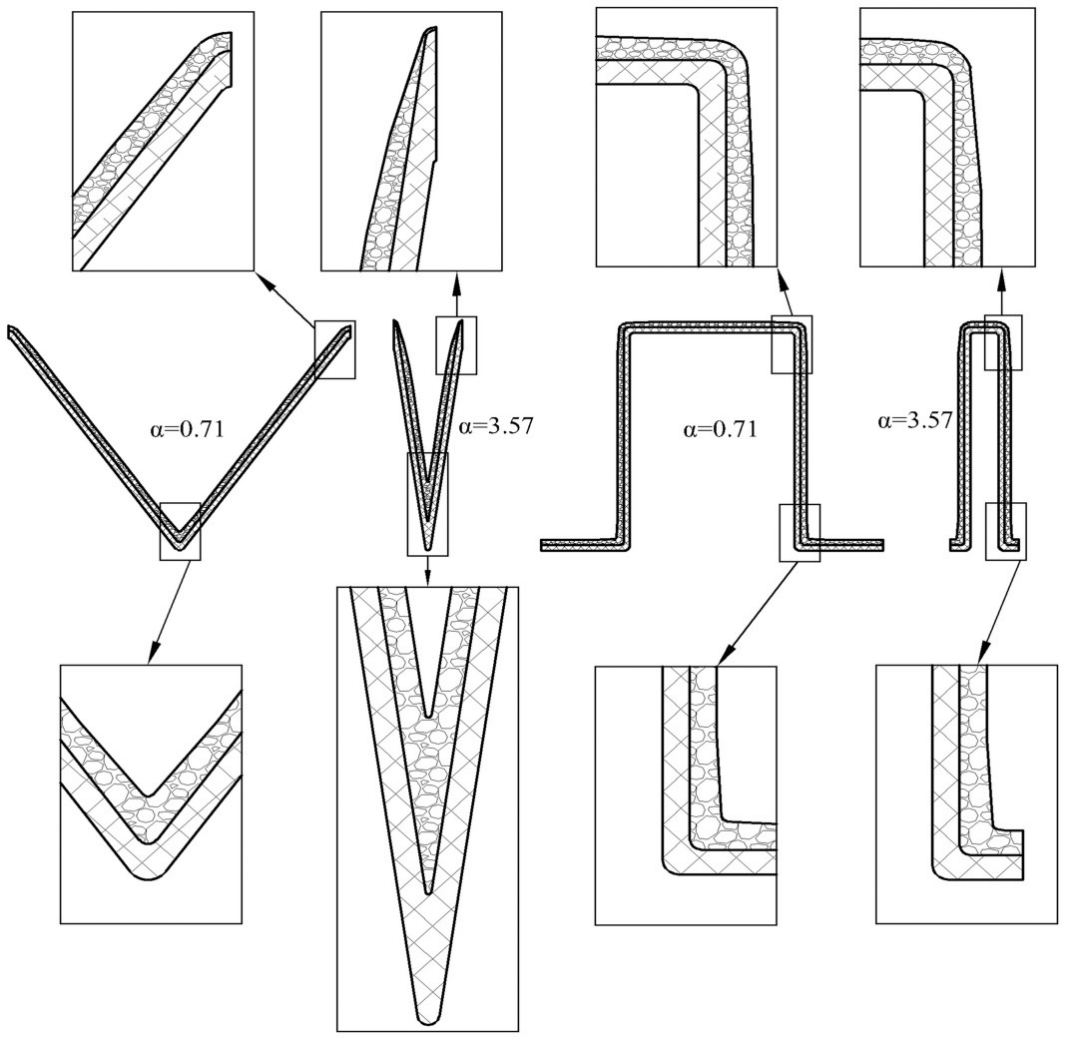
Unevenness of dust deposition when *W* equals 30 mg/cm^2^.

Figs 16 and 17 present the changes in the NAVFs of the V-shaped and U-shaped filters during dust loading, respectively. Only the variation of the NAVFs under the *W* values of 0, 10, 20 and 30 mg/cm^2^ are drawn for the purpose of ensuring clarity of the figures. As illustrated in Fig 16, the NAVF varies substantially at the upper and lower pleated corners, but remains basically constant in the middle region. Specifically, as α and *W* grow, the NAVF at the upper pleated corner rises while that at the lower pleated corner falls. In addition, the larger α and *W* are, the larger the NAVF variation in the pleated area is. This phenomenon is caused by the uneven deposition of dust. The larger α and *W*, the greater the variability of dust cake thickness at the upper and lower pleat corners (Fig 18).

Fig 17 depicts the variations of the NAVFs on the top, bottom and flank filter surfaces of the U-shaped filter during dust loading. The NAVFs varies greatly in the pleated area, while that is basically constant in other regions. As α and *W* grow, the NAVF in the upper pleated area increases, whereas that in the lower pleated area decreases, exhibiting the same trend as the V-shaped filter. The decrease in the NAVF in the lower pleated area results from the deposition of dust, while the increase in the NAVF in the upper pleated area arises from the small dust cake thickness. Meanwhile, this phenomenon is more serious under a larger α, as shown in Fig 18.

**Fig 19:**
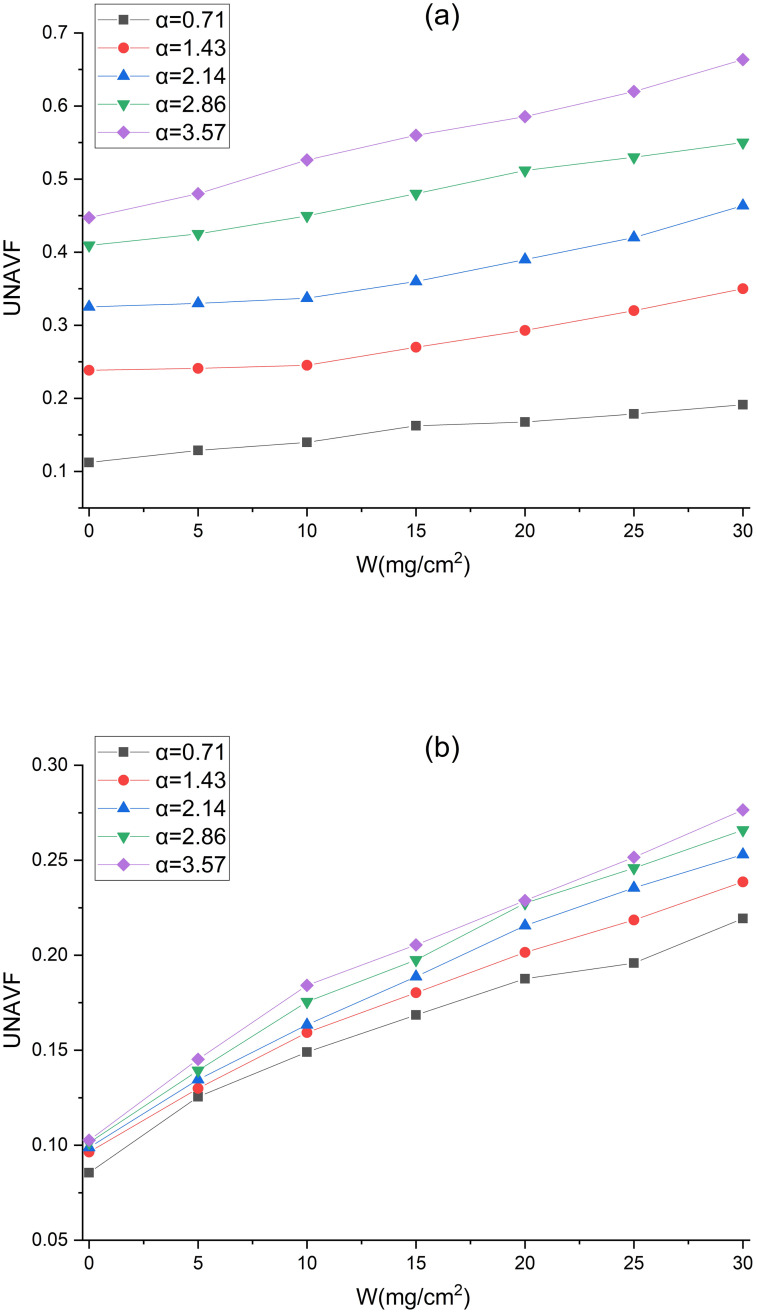
Changes in the NAVF with *W* (a) V-shaped; (b) U-shaped.

To quantitatively investigate the impact of dust deposition on the unevenness of NAVF (UNAVF), the relative root mean square error of the NAVFs is adopted in this study for evaluation [16]:

$$UNAVF=\sqrt{\frac{\sum_{1}^{N}{\left(\nu -\bar{\nu }\right)}^{2}}{N-1}}/\bar{\nu }$$ (9)

where *v* is the NAVF at each monitoring point (cm/s), N is the number of monitoring points, $\bar{\nu }$ is the average NAVF at the monitoring points (cm/s). The variation of the UNAVF with *W* is depicted in Fig 19. The UNAVF ranges from 0.1123 to 0.6635 and from 0.0855 to 0.2765 for V-shaped and U-shaped filters, respectively. The UNAVFs of V-shaped and U-shaped filters change in the same trend, both increasing with the rise of α and *W*. This phenomenon can be explained by the difference in the pleated geometry and the uneven deposition of dust particles. It can also be seen from Fig 19 that the U-shaped filter has a smaller UNAVF than the V-shaped filter under the same α and *W*, which is consistent with the law of the total pressure drop variation in Fig 14. Under the same α and *W*, the lower the UNAVF, the smaller the total pressure drop. As a result, the law of the total pressure drop variation can be judged by UNAVF. In conclusion, the U-shaped filter is recommended because it boasts a smaller pressure drop and a longer replacement cycle provided that other conditions are the same.

## 4 Summary and conclusions

In this study, a numerical simulation method for dust particle deposition on the V-shaped and U-shaped filters at small Stokes numbers was presented. In particular, the suitability of the model was verified by means of the HWA to generate local quantitative air velocity data and the simulated pressure drop was verified experimentally. In the simulation of PM_10_ loading, the dust cake thickness was considered to be proportional to the NAVF due to the small Stokes number of the particles in the experiment.

By comparison, the relative mean deviations of experimental and simulated total pressure drops are 3.12% and 1.19% for V-shaped and U-shaped filters, respectively. The pressure drop during dust loading can be accurately predicted by numerical simulation. The simulation method can save a significant amount of CPU time compared to the previous method of modelling the growth of dust cake by defining user defined functions. Furthermore, it was found that under the same α and *W*, both the pressure drop and the NAVF of the U-shaped filter were lower than the V-shaped filter. Therefore, the simulation method can be used to serve as a reference for the optimization of the pleated structure of the filter so as to reduce the resistance.

## Supporting information

S1 Data (XLSX)
